# Miscellaneous Surgical Essays

**Published:** 1781-04

**Authors:** 


III. *Vermiſchte Chirurgiſche ſchriften, herauſgegeben von* Johann. Leberecht Schmucker, *Königl. Preuſſiſchen erſten General Chirurgus von der Armee, Director der Chirurgiſchen Kriegſlazarethe und Mitglied der Kais. Acad. der Naturforſcher.* i. e. *Miſcellaneous Surgical Eſſays publiſhed by* John Leberecht Schmucker, *Firſt Surgeon General of the Army, and Director of all the Military Hoſpitals in Pruſſia,* &c. 8vo. Berlin. vol. 1. 352 *Pages, with Copper Plates.*

WE have here a valuable collection of original ſurgical eſſays, ſome of them the productions of the editor himſelf, and all of them vouched by him as authentic; we ſhall review them in the order in which they are publiſhed.

I. *On the Amputation of Extremities,* by Mr. Schmucker.——The author begins with a ſhort hiſtory of this operation, ſhewing how cruel and defective it was formerly, and how mild and how much improved it is at preſent. He does not, like like M. Bilguer, reject amputation entirely, but recommends it in many cafes.

The firft cafe in which he advifes it, is in that of a frozen limb become gangrenous from a too fudden application of heat. In cafes of this fort, we are told, that the mortification fpreads with fo much rapidity, that fcarifications, and all other remedies, are incapable of reftoring the loft feeling, fo that the patient has no chance of life but from amputation. In fuch fituations, however, we think the knife ought not to be ufed too precipitately: we fhould wait to fee whether nature will not with lefs danger, though perhaps more flowly effect what we intended to do more fpeedily, but with infinitely greater rifk to the patient.

Speaking of mortifications from internal caufes, Mr. Schmucker obferves, that we ought not to amputate till the gangrene has fixed its limits, and there is no poffibility of faving the limb, and that it fhould then not be deferred. But here too, in our opinion, the furgeon fhould wait to fee whether nature will not throw off the mortified part.

He thinks that caries arifing from internal caufes, and not yielding to milder remedies, requires amputation, the complaint being in general, tal local, and not always accompanied with a vitiated ſtate of the habit, as ſome have ſuppoſed. He deſcribes ſeveral caſes in which the operation ſucceeded. In one of theſe there was a caries of the foot, which came on after venæſection, though no tendon or nerve ſeemed to have been wounded by the lancet. For the ſpace of two years the patient ſuffered moſt excruciating pain, and a variety of remedies having proved ineffectual, amputation was had recourſe to and ſhe recovered. In another ſimilar caſe that ſeemed to take its riſe from a ſlight contuſion, the amputation proved ſucceſsful, although the patient was already much reduced by a hectic fever.

In treating of wounds and contuſions of the extremities, our author has attempted to aſcertain from his own experience the caſes that require amputation.

He obſerves, that violent contuſions of the toe ſoon terminate in gangrene; that gun-ſhot wounds of the tarſus are always dangerous; but that if a ſmall ball paſſes through this part, the danger is much leſs than if it remains in it, becauſe in the latter caſe the uſual conſequences are violent inflammation, locked jaw, and gangrene; the ball changes its ſhape, and being extremely difficult to extract, amputation becomes the only remedy that can ſave the patient. The os calcis, he ſays, may be ſhot through without any great danger, but if the tarſus, together with the articulation and one or both *malleoli* are injured by a cannon ball, and the tendons and ligaments lacerated; or if the knee joint is wounded in a ſimilar manner, amputation becomes abſolutely neceſſary in each of theſe caſes.

Mr. Schmucker obſerves, that gun-ſhot wounds of the tibia, the fore-arm, and even the arm, ſeldom require amputation, the bones of theſe parts being ſo ſituated, that the ſplintered portions of the bone may be eaſily removed. He ſpeaks of wounds of the os femoris as the moſt dangerous of any, on account of the hardneſs and fragility of this bone, and its being eaſily ſplintered, as well as the injury uſually done to ſeveral large arteries. If the wound is only a little above the knee, where there are but few muſcles, he thinks the neceſſity for amputation will be leſs preſſing than if the wound is higher up in the thigh. When the neck and condyles of the os femoris are wounded, he thinks there remains but little chance for the patient's recovery, as the amputation of the thigh at the articulation is ſo dreadful, difficult, and dangerous, that it never can ſucceed. This opinion, however, ever, ſeems to be not well founded. An account has lately been publiſhed in the Edinburgh Medical Commentaries of this operation, as performed at Northampton, from which it appears, that the death of the patient on the 18th day after the amputation was owing to the previouſly diſeaſed ſtate of the parts, rather than to any immediate effects of the operation; and Mr. Kerr, the author of the paper in queſtion, is ſo much convinced of the expediency of the operation in certain caſes, that "in ſuch," he ſays, "he ſhall "not heſitate to perform it when they occur."

Mr. Schmucker obſerves, that wounds of the carpus and metacarpus ſeldom require amputation, becauſe we are here able to make the neceſſary openings with eaſe; but that when the contuſion is very conſiderable, and the tendons and ligaments are much lacerated, the operation becomes neceſſary.

He is averſe to performing the operation in the articulation of the knee, cubitus, or carpus, becauſe the ſtumps can never be covered with fleſh, and aponeurotic and membranous parts do not readily ſuppurate.

In amputating the fingers or toes, he conſtantly makes uſe of a biſtoury, and ſeparates them at the joints, taking care to ſaw off the cartilages

 of of the phalanges, becauſe they exfoliate with difficulty, and retard the cure ſeveral weeks.

His method of preventing hæmorrhage after amputation, is by applying doſſils of fine lint, of the thickneſs of half an inch, to the mouths of the veſſels, and the conſtant preſſure of a hand on the ſtump during at leaſt four-and-twenty hours. He does not truſt to this method, however, for ſecuring the femoral artery, but prefers the ligature for that purpoſe. He tells us, he has often ſeen the doſſil of lint fall off at the firſt dreſſing, and yet no hæmorrhage has followed.

In amputations he applies a narrow riband round the limb, above the inciſion, and cuts through the integuments and muſcles with a ſingle inciſion, a mode of operating by-the-bye, that is now pretty generally and deſervedly exploded. The knife he recommends is ſlightly curved, and ſmaller than that commonly uſed. Inſtead of Gooch's retractor, he uſes a piece of parchment, eighteen inches long, and ſix broad, for the purpoſe of drawing back the muſcular fleſh, while he ſaws through the bone. The ſtump being covered with lint, the integuments are drawn downwards and ſecured by ſlips of ſticking-plaſter, after which the whole is covered with with a hog's bladder, which he prefers to the cap commonly employed, as it will ſerve to prevent ſlight hæmorrhages. He applies his bandage, after the manner of M. Louis, from above downwards.

II. *An Hiſtorico-practical Eſſay on the medical Uſe of Leeches, by* the ſame.—This paper is illuſtrated by a coloured engraving of the *hirudo medica*, of which an exact deſcription is given. We are told, that it ſhould be taken from pure limpid rivers that have a ſandy bed, as the leeches caught in ſtagnant waters occaſion ſwelling and inflammation. In general, thoſe which have been the moſt recently taken bite the quickeſt.

Our author ſpeaks of their ſalutary effects in ophthalmia, after repeated venæſections had proved ineffectual. In a violent cephalalgia, owing to an increaſed determination to the head, he has ſeen ten or twelve leeches applied to the temples, procure an almoſt immediate relief. He aſſures us, that an inflammatory ſore throat, attended with very difficult deglutition and breathing, will oftentimes yield to ſixteen or twenty leeches applied to the throat and behind the ears, more ſpeedily and with greater certainty than to repeated bleedings. In the toothach, ach, when not owing to a caries, he has found leeches applied to the gums extremely ſerviceable.

He aſſerts, that in a pleuriſy twelve leeches applied to the ſide affected, will prove more efficacious than a bliſter. He has ſeen hæmoptyſis, from obſtructed hæmorrhoids or menſes, ſtopped by applying leeches to the anus; and in the generality of caſes, they have been the means of reſtoring the latter evacuation.

In the piles their uſe is well known; but Mr. Schmucker obſerves, that in ſuch caſes they are ſerviceable only where the ſwelling is not larger than a hazel nut. To prevent a return of the tumour, he recommends the application of cold water to the part, or a cold glyſter morning and evening when the ſwelling is internal. When the tumour is of conſiderable bulk, and its membranes of courſe are much thickened, he finds them of no uſe.

In retention of urine he has often experienced the good effects of ſix leeches applied to the perinæum; and we are farther told, that four of them applied to the end of the finger in a beginning paronychia, generally remove the complaint.

III. *A Deſcription of a very ſimple Machine for Fractures*

*Fractures of the Os Femoris, by* J. A. Theden, *Surgeon-General to the Prussian Army.*——The machine here described, and of which an engraving is added, differs but little from the splints that are now generally used in fractures of the thigh in this country. It consists of two splints, the outermost of which is perforated at each end, where it presses on the great trochanter of the os femoris, and on the eminences of the knee; and the inner splint is broad at its upper extremity, and formed into a crescent, to prevent it from pressing on the pubis and os ischium.

IV. *On the Use of Assafœtida in carious Ulcers, by* Mr. Block. The author having tried madder-root without effect in cases of this sort, had recourse to assafœtida; a medicine, the efficacy of which has lately been extolled by Mr. Theden, in obstructions of the abdominal viscera. Mr. Block mixes an ounce of assafœtida with half an ounce of *conchæ preparatæ*, and half a drachm of camphor; and of this powder he prescribes from a scruple to half a drachm twice a day. Externally he applies only dry lint. Four cases are related in which it had proved serviceable, by changing the ichorous discharge from the sore into a laudable pus.

We

We have had occaſion to notice a ſimilar effect from myrrh.

V. *On Herpes, Melancholia, and Palſy, by* Mr. Evers.—Theſe obſervations were made in a marſhy country, where the herpes, we are told, is a very common complaint; and ſeems to be generally owing to a diſeaſe of the liver, or to obſtructions of the meſenteric glands. Sometimes, however, the author has had reaſon to aſcribe them to obſtructed evacuations, or to a ſcorbutic or venereal acrimony. The greater number of the patients whom he has had occaſion to ſee labouring under obſtinate herpes, were pale, yellow, and emaciated. In one caſe, the *pilul. ſcill. c.* of the *Pharm. Edin.* with occaſional purgatives, effected a cure; in another, the diſeaſe yielded to the uſe of Plumer's pill; in a third, our author ſucceeded by preſcribing Seltzer water, a milk diet, and ſixty drops of the eſſence of white pimpernel every evening; and in a fourth, the complaint was relieved by ſixty drops of the acid of ſalt, taken for ſome time four times a day.

In melancholia, when the patient's coſtiveneſs, the fulneſs of the abdomen, and a ſmall, ſlow pulſe ſeemed to indicate that obſtructions in the belly were the cauſe of the diſorder, our author gave gave the belladona with fuccefs. Five grains of the leaves of the plant were adminiftered every morning, combined with an equal quantity of rhubarb, and occafionally the patient took a purgative medicine. In the greater number of cafes, the cure was effected by thefe means in five weeks.

In paralytic affections, when the patients were pale and emaciated, complained of coftivenefs and had a flow pulfe, our author, after flightly purging them, gave the fame medicine, the belladona, increafing the dofe of it by degrees to ten grains daily, and combining it, as in melancholia, with rhubarb.

VI. *An Account of a Suppuration of the Omentum, by* M. Bingers.—The difeafe here defcribed was occafioned by a contufion on the gaftric region. The violent pain, of which the patient complained at firft, gradually went off, but he ftill continued to feel an obtufe pain, which after fome time was accompanied with lofs of appetite, difturbed fleep, and frequent vomiting. Thefe fymptoms continued during a whole year, when a tumour began to form, which at length fuppurated, and afforded a confiderable difcharge of pus, intermixed with portions of the omentum. The wound healed in about

 fix

ſix weeks, and left no other complaint but a hernia.

VII. *Of a Wound happily healed*, by the ſame. —In this caſe, the patient was wounded by a ſword which paſſed tranſverſely through the abdomen, from near the vertebræ lumborum to the left ſide of the navel. The fæces were diſcharged through both wounds in ſuch quantity, that the natural evacuation by the anus was for ſome time entirely ſuſpended. In this ſtate the wounds were kept open, clyſters were frequently thrown up, and the patient was confined to a light liquid diet. By theſe means the diſcharge of fæces through the wounds gradually leſſened, and they were at length wholly evacuated at the anus, ſo that at the end of ſix weeks the patient was perfectly recovered.

VIII. *On the internal Uſe of Lime Water and Soap in a Caſe of Calculus*, by the ſame.—In the caſe here related, the patient took ten ounces of lime water and a drachm of ſoap twice a day, and at the end of a fortnight, the ſymptoms, which were at firſt very violent, entirely ceaſed. The ſtone made its way into the urethra, from whence our author extracted it. He ſuppoſes that it was leſſened by the ſolvent powers of the remedy; but we are by no means convinced that that this idea is well founded. He adds, that in nephritic complaints in children he has often ufed this medicine with fuccefs.

IX. *An Inftance of the Inteftines being perforated by Worms*, by the fame.—In this cafe the patient was attacked with fymptoms of fever, and a painful fwelling appeared near the pubis, which at length fuppurated. A confiderable quantity of pus and four lumbrici were difcharged by this abfcefs, which healed without difficulty.

X. *Of a Tumour near the Rectum*, by the fame. —The tumour here fpoken of, after remaining indolent during fix years, at length became painful and fuppurated. On dilating the abfcefs, a ftone was difcovered of the fize and fhape of a cherry-ftone, contained within a hard cyft, which was removed by the knife.

XI. *Of the Cure of a Venereal Fiftula Urinaria*, by the fame. —Four cafes of this kind are related. In one of thefe Mr. Bingers fucceeded, by introducing bougies into the urethra, and applying compreffes to the opening in the perinæum. In another cafe, which was cccafioned by an ill-treated gonorrhœa, he dilated the opening in the perinæum, drew the lips of the wound together by means of fticking-plafter, and in feven

 days days the cure was completed. In a third cafe, attended with confiderable indurations, bougies introduced into the urethra excited a fuppuration, and by that means removed the hardnefs. Compreffes were applied externally, as in the former cafe, and the patient obtained a cure. In a fourth cafe, he met with more difficulty. The exterior opening was very fmall, but internally there was a large fac extending from the anus to the fcrotum, and the urine by remaining in this bag had by its irritation occafioned a fiftula that extended to the penis. Dilatation of the orifice, and even deeper incifions, compreffes, and bougies, all proved equally inefficacious; but at length by introducing a catheter into the urethra, and the patient's wearing it conftantly, a cure was effected.

XII. *Of a flefhy Excrefcence in the Rectum*, by the fame.—In this cafe, the patient was much emaciated by a long continued and profufe evacuation of bloody water by ftool, a *procidentia recti* at length took place, and a flefhy excrefcence of a dark red colour, and of the fize of a man's fift, was found adhering to the inteftine by a fhort thick pedicle; it bled much, and occafioned violent pains and fpafms in the abdomen. It being found impoffible to remove it all at at once, it was brought away in detached portions, and all the bad ſymptoms diſappeared; but ſoon after, a ſecond excreſcence, ſimilar to the former one, protruded at the rectum, and being removed by ligature, the patient was completely cured.

XIII. *Caſe of a cancerous Teſticle*, by the ſame. —The teſticle here ſpoken of was enlarged to the ſize of a man's fiſt, and was accompanied with ſeveral cancerous excreſcences and a fiſtulous opening that afforded a putrid ichorous diſcharge. Our author extirpated it with ſucceſs, although the ſpermatic chord was thickened to the ſize of the thumb, even beyond the abdominal ring. The chord was divided, and a ligature paſſed round it below the ring.

XIV. *On the Uſe of cold Water in Wounds of the Joints*, by the ſame. Two wounds into the cavity of the knee-joint, one by a ſword, and the other by a piece of glaſs, were both healed without any alarming ſymptom by the external application of cold water. In both caſes the lips of the wounds were drawn together, and covered by emplaſtrum adhæſivum, blood was drawn from the arm, and compreſſes dipped in cold water were applied to the knee, and renewed as often as they they began to grow warm, till the cure was completed.

[ *To be continued.* ]

