# Errata

**Published:** 1781-06

**Authors:** 


					p. 240, 1. 20, for white pimpernel read white bumet
faxifrage.-

				

